# Immobilized *Staphylococcus aureus* phospholipase C on calcium alginate-chitosan: improved performance and industrial potential in soybean oil refining

**DOI:** 10.3389/fbioe.2025.1706906

**Published:** 2025-11-07

**Authors:** Areej Ali Alzahrani, Najeh Krayem, Mona Alonazi, Eman Al-Shehri, Habib Horchani, Abir Ben Bacha

**Affiliations:** 1 Department of Biochemistry, College of Science, King Saud University, Riyadh, Saudi Arabia; 2 Laboratory of Biochemistry and Lipase Enzyme Engineering, ENIS, University of Sfax, Sfax, Tunisia; 3 Science Department, College of Rivière-Du-Loup, Rivière-DuLoup, QC, Canada

**Keywords:** phospholipase C, *Staphylococcus aureus*, enzyme immobilization, calcium alginate-chitosan, enzyme stability, soybean oil degumming, reusability

## Abstract

Phospholipases are versatile biocatalysts with wide applications in the food and oil industries due to their ability to hydrolyze phospholipids and improve product quality. In this study, production of an extracellular phospholipase C from *Staphylococcus aureus* (PLC_S.a_) was optimized, purified, and its immobilization on calcium alginate-chitosan (CAC) was evaluated for soybean oil degumming. Statistical optimization significantly enhanced PLC_S.a_ production, showing maximum yields during the exponential growth phase (232.5 U/mL at 34 h) and optimal activity at 37 °C and pH 7.5. Medium composition remarkably influenced enzymatic activity, with glucose (2%), bovine serum albumin (BSA, 1.5%), Tween 20/80 (0.5%–1%), and Zn^2+^/Ca^2+^ (0.1%–0.3%) identified as the most effective enhancers. Immobilization markedly improved enzyme stability, thermal and pH tolerance, and catalytic efficiency compared to the free form. PLC_S.a_ exhibited broad substrate specificity, showing maximum activity toward phosphatidylcholine, and maintained activity in the presence of bile salts and divalent cations. Biotechnological application demonstrated efficient reduction of phosphorus levels in soybean oil from 198 mg/kg to below 2.5 mg/kg within 10 h. Reusability assessment showed that immobilized CAC-PLC_S.a_ retained 100% activity for two cycles, 85.5% after five cycles, and 60% after nine cycles, confirming its operational stability. These results highlighted the potential of immobilized PLC_S.a_ as a cost-effective, eco-friendly, and reusable biocatalyst for large-scale vegetable oil refining.

## 1 Introduction

Phospholipids form the main structural elements of cellular membranes across all organisms. Their hydrolysis is carried out by phospholipases, enzymes with either acylhydrolase or phosphodiesterase activity (Cerminati et al., 2019). These enzymes are broadly distributed, contributing to microbial virulence and nutrient acquisition (Strahl and Errington, 2017), while they play crucial roles in growth, lipid metabolism, cell migration, membrane dynamics, signaling, and programmed cell death in eukaryotes (Tracey et al., 2018; Yang et al., 2018).

Phospholipases occur as secreted, membrane-bound, or intracellular enzymes, activated at aqueous–organic interfaces. They are grouped according to their cleavage site: PLA_1_/PLA_2_ remove fatty acids at sn-1 or sn-2, PLB hydrolyzes both, PLC releases diacylglycerol and phosphorylated heads (phosphatidylcholine (PC)- or phosphoinositide (PI)-specific), while PLD generates phosphatidic acid and can catalyze transphosphatidylation (Cerminati et al., 2019).

Enzymes are increasingly replacing chemical processes due to their specificity and eco-friendly conditions, supporting white biotechnology goals of sustainable production. Advances in molecular tools and protein engineering now enable efficient discovery, optimization, and large-scale enzyme applications (Bornscheuer, 2018). Among enzymes, phospholipases attract growing interest for industrial use, particularly in food and pharmaceuticals. In food processing, they enhance emulsifying properties in baking, dairy, and egg-based products and play a major role in vegetable oil degumming to improve yields (Cerminati et al., 2019).

Crude vegetable oils like soybean, rapeseed, and sunflower contain mainly triglycerides (∼98%) along with minor bioactive compounds such as tocopherols, sterols, and polyphenols that benefit health (Gharby, 2022). They are also a complex mixture of phospholipids (1.75%–3%), sterols, pigments, tocopherols, and free fatty acids, which can form gum deposits during storage and cause problems in food applications. Phospholipids in particular contribute to dark coloration and off-flavors (Borrelli and Trono, 2015). Therefore, degumming is a crucial refining step that removes these gums, mainly phospholipids, thereby improving oil quality (Elena et al., 2017). For effective phospholipid removal, the oil’s phosphorus content should be reduced to below 10 mg/kg (Dos Passos et al., 2022). Common methods for phospholipid removal include water and acid degumming: water degumming targets hydratable phospholipids, while acid degumming, using acids such as phosphoric or citric acid, removes non-hydratable phospholipids (Alonazi et al., 2023; Dos Passos et al., 2022). These conventional degumming methods do not always yield high-quality oil (Sampaio et al., 2019). Water degumming removes only hydratable phospholipids, while acid and super degumming eliminate only part of the non-hydratable phospholipids. Moreover, traditional degumming cannot consistently reduce phosphorus below the <10 mg/kg required for further refining (Sampaio et al., 2019).

In recent years, enzymatic degumming has gained considerable attention, with some studies progressing to pilot-scale applications. This emerging technique uses phospholipases to remove phospholipids from crude oils, converting non-hydratable phosphatides into a hydratable form. As a result, enzymatic degumming offers a safe, biologically based, and environmentally friendly alternative for oil refining (Sampaio et al., 2019; Cerminati et al., 2019).

Several phospholipases are used in oil degumming, including LysoMax^®^ (PLA_2_) and Lecitase^®^ Ultra (PLA_1_) from *Thermomyces lanuginosus* (Lamas, 2022). Phospholipase C (PLC) is preferred as it hydrolyzes the phosphodiester bond of phospholipids, generating sn-1,2-diacylglycerols (DAGs). These DAGs not only improve oil quality but also provide essential fatty acids, including linoleic, α-linolenic, and oleic acids, which support ω-6/ω-3 biosynthesis and cardiovascular health (Xiang et al., 2020).

PC-PLCs hydrolyze phosphatidylcholine PC and phosphatidylethanolamine PE, while PI-PLCs target phosphatidylinositol PI, but engineered PLCs can now hydrolyze PC, PE, and PI without specificity, ensuring complete phospholipid removal and higher oil yield. For example, PLC-Y from *Bacillus cereus* and LS PI-PLC from *Lysinibacillus sphaericus* efficiently degum soybean oil and can be produced cost-effectively at an industrial scale (Cerminati et al., 2019).

Most PLCs used in degumming are free enzymes that can be used only once, increasing operating costs. Immobilized enzymes are favored over their free forms because they offer higher stability and can be easily separated from reaction mixtures and reused multiple times, thus reducing operating costs. In addition, immobilization on appropriate supports enhances structural integrity, protects the enzyme in harsh conditions, improves thermal and pH stability, and can fine-tune activity, specificity, and selectivity, making them more suitable for industrial applications (Cerminati et al., 2017; Luo et al., 2023). Common immobilization methods include entrapment in calcium alginate (CA), covalent binding to chitosan or gelatin, and crosslinking with glutaraldehyde, or combinations thereof, with CA, chitosan, and gelatin being the most popular polymers (Yu et al., 2012; Yu et al., 2022). For example, lecitase (PLA_1_) immobilized in gelatin crosslinked with glutaraldehyde efficiently degummed rice bran oil and retained activity after six cycles (Sheelu et al., 2008). WaPLA_2_-I and WaPLA_2_-II from *Walterinnesia aegyptia* venom, immobilized on calcium-alginate-chitosan (CAC) beads, showed the highest soybean oil degumming yields (85%–87%), retained over 50%–60% activity after eight recycles or 120 days at 4 °C, and reduced residual phosphorus from 168 mg/kg to <10 mg/kg in 4 h, outperforming CA and CA-gelatin supports (Bacha and Alonazi, 2024).

Chitosan and CA are among the most widely used polymers for enzyme immobilization due to their biocompatibility and stability. Chitosan, a flexible biopolymer derived from chitin, provides strong physical and ionic interactions with enzymes, improving adsorption and activity. When combined with CA, it increases mechanical strength and resistance to harsh conditions, making the immobilized enzymes more durable and suitable for repeated industrial use (Weining et al., 2022).

Microbial PLCs from *Bacillus cereus* (Ravasi et al., 2015; Jiang et al., 2015), *Thermococcus kodakarensis* (Marchisio et al., 2022), *L. sphaericus* (Cerminati et al., 2017), and *Bacillus stearothermophilus* (Alonazi et al., 2023) have been widely studied for oil degumming due to their high activity and relatively simple production. However, most of these enzymes are used in free form, limiting reusability and increasing costs.

*Staphylococcus aureus (S. aureus)* is a common bacterium found in diverse environments. In our laboratory, it has been shown to produce extracellular enzymes such as lipase and protease (Alonazi, 2024; Bacha et al., 2018) but remains largely unexplored as a source of industrial PLC compared to well-characterized PLCs. Its ability to produce multiple enzymes makes it a promising candidate for developing enzymatic cocktails for various industrial applications. To address this limitation, we investigated the production optimization, the immobilization of *S. aureus* PLC (PLC_S.a_) on CA-chitosan (CAC) beads and assessed its activity, stability, and reusability, providing insights into its potential for large-scale soybean oil degumming.

## 2 Materials and methods

### 2.1 Materials and reagents

The chromatography material (G-75 Chromatography (2 × 100 °Cm), Mono Q-Sepharose), SDS-PAGE technique, pH-stat, and rotary shaker were obtained from Bio-Rad (USA). The automated Edman degradation method was performed using a PROCISE instrument (Applied Biosystems, Foster City, CA).

Chemicals were obtained from commercial sources. Glucose, lactose, sucrose, maltose, dextrin starch, peptone, tryptone powder, beef extract, yeast extract, BSA, KH_2_PO_4_, NaCl, Triton X-100, Tween 20, Tween 80, NaDC, NaTDC, BaCl_2_, BaSO_4_, CaCl_2_, COCl_2_, CuSO_4_, MgCl_2_, FeCl_3_, ZnSO_4_, ZnCl_2_, CaCO_3_, Celite, silica, CA, chitosan, and glutaraldehyde were acquired from Bio-Rad (Hercules, CA, USA). Phospholipase substrates PC, PE, PI, PG, PS, cardiolipin, and sphingomyelin (SM) were purchased from Sigma-Aldrich (St. Quentin-Fallavier, France). Sodium dodecyl sulfate (SDS), acrylamide, ammonium persulfate, N,N,N′,N′-tetramethyl ethylenediamine (TEMED), β-mercaptoethanol, Coomassie brilliant blue R-250, and protein markers for molecular mass were also provided by Bio-Rad (Hercules, CA, USA).

### 2.2 PLC production and optimization of medium culture composition

The *S. aureus* strain ALA1 (GenBank accession no. KF678862) was originally isolated and characterized by Ben Bacha et al. (2016).

Prior to fermentation, the *S. aureus* strain ALA1 was precultured in nutrient broth overnight at 37 °С shaking flasks at 200 rpm. Then, the pre-culture was inoculated in a 1 L culture shaking containing 100 mL of TSB growth medium at 37 °C and 200 rpm. Using “one variable at a time,” several inoculated growth media were maintained with different carbon (glucose, lactose, sucrose, maltose, dextrin and starch) or nitrogen (peptone, tryptone powder, beef extract, yeast extract, BSA, KH2PO4) sources at various concentrations (0.5%–3%), various metal ions (BaCl_2_, BaSO_4_, CaCl_2_, COCl_2_, CuSO_4_, MgCl_2_, FeCl_3_, ZnSO_4_, ZnCl_2_, CaCO_3_) at 1% concentration, or various surfactants (Triton X-100, Tween 20, Tween 80 (0.25–1.5%), NaDC and NaTDC (0.1%–0.5%)), then at different incubation times (0–96 h), various temperatures (20 °C–45 °C), and pH (6.0–9.0).

Bacterial growth was monitored by measuring the optical density at 600 nm, with samples collected every 4 h and centrifuged at 10,000 × g for 10 min. The resulting supernatants were considered crude enzyme extracts and subsequently used for activity assays. Subsequently, optimal phospholipase production was evaluated using the pH-stat method with PC as substrate under standard conditions.

### 2.3 PLC_S.a_ purification

In order to produce PLC_S.a_, *S. aureus* strain ALA1 was inoculated in the TSB growth medium enriched with 2% glucose, 1.5% BSA, 1% Tween 20, 0.3% NaDC, and 0.2 g/L ZnCl_2_ with an initial OD of 0.2 at 600 nm and pH 7.0. After 34 h, bacterial cells were discarded by centrifugation (10,000 × g, 30 min) from 100 mL. The obtained crude phospholipase solution (96 mL) was initially subjected to ammonium sulfate (NH4)_2_SO_4_ fractionation (30%–70%), followed by Sephadex G-75 gel filtration and finally, the Mono Q-Sepharose ion exchange chromatography step. After confirming the absence of unbound proteins by washing with buffer A (25 mM Tris-HCl buffer containing 25 mM NaCl and 2 mM benzamidine, pH 8.0), the retained proteins were released through a linear NaCl gradient (25–300 mM) in buffer A, applied at a flow rate of 0.5 mL/min, with eluates collected in 2-mL fractions.

### 2.4 Protein analysis

Protein concentration, molecular mass, and purity of the PLC_S.a_ were assessed using the Bradford assay and SDS-PAGE on 15% polyacrylamide gels under reducing conditions (Bradford, 1976; Laemmli, 1970). The N-terminal sequence of the native PLC_S.a_ was determined through automated Edman degradation with a PROCISE sequencer (Applied Biosystems, Foster City, CA; Hewick et al., 1981). The molecular mass of the pure PLC was then accurately determined on a Voyager DE-RP MALDI-TOF mass spectrometer (Biosystem, Framingham, MA, USA). Mass spectra recorded in linear mode were externally calibrated with suitable standards and analyzed by the GRAMS/386 Software.

### 2.5 PLC_S.a_ activity measurement and substrate specificity

Phospholipase activity was assayed using the pH-stat titrimetric method described by Zwaal et al. (1971). The reaction mixture contained a 5 mM phosphatidylcholine (PC) emulsion as substrate, supplemented with 6 mM sodium deoxycholate (NaDC) and 0.5 mM ZnCl_2_, and was carried out at 50 °C and pH 9.0. Under these standard conditions, one unit of activity corresponded to the release of 1 μmol of phosphocholine per minute.

The activity of PLC_S.a_ was also evaluated on various phospholipid substrates, including PE, PI, PG, PS, cardiolipin, and sphingomyelin, to assess its substrate specificity. Activities are expressed as percentages relative to the activity on PC as described above.

### 2.6 PLC_S.a_ immobilization

After 34 h of *S. aureus* incubation in the appropriate medium at 37 °C on a rotary shaker at 200 rpm, the crude enzyme solution obtained after centrifugation (30 min at 10,000 x g) was subjected to an ammonium sulfate fractionation (30%–70%). Then, the precipitate was resuspended in buffer A. The collected PLC solution after centrifugation at 10,000 × g for 30 min was kept at 4 °C.

Immobilization of PLC_S.a_ on silica gel, CaCO_3_, and Celite 545 was performed following the procedure of Rosu et al. (1997). Two grams of each support was combined with 5 mL of PLC solution and incubated at 4 °C for 2 h under continuous stirring at 200 rpm. For CA entrapment, the method of Ateş and Mehmetoğlu (1997) was applied with slight modifications: 5 mL of PLC solution was incorporated into 50 mL of sodium alginate (2.0%) prepared in 5.0% acetic acid under agitation. The mixture was extruded dropwise (5 drops/s) through a 10-mL syringe needle into 0.2 M CaCl_2_, generating beads of approximately 2 mm in diameter. Chitosan-alginate-CaCl_2_ (CAC) beads were obtained using a modified protocol by Sun et al. (2005), where 5 mL of the enzyme solution was blended with 25 mL of chitosan (4%) dissolved in 0.2 M CaCl_2_/acetic acid (5%), followed by dropwise addition of 25 mL of sodium alginate (4%) in 5% acetic acid to induce bead formation. For alginate–gelatin (CAG) entrapment, a slightly modified procedure of Zhu et al. (2004) was used: 25 mL of gelatin solution (8%) was mixed with 25 mL of sodium alginate (4%) in 5% acetic acid, then 5 mL of PLC solution was incorporated, and 0.2 M CaCl_2_ was gradually added under constant stirring (200 rpm) to form beads. All beads were subsequently hardened with glutaraldehyde, thoroughly washed, and freeze-dried.

The immobilization efficiency was determined by comparing the residual activity of immobilized PLC to the initial activity of the free enzyme, using the following equation:

$$\text{Residual Activity }\left(\%\right)=\left(\text{Initial free enzyme activity}/\text{Activity after immobilization}\right)\times 100$$

The particle or bead sizes of the supports were as follows: Celite 545 particles typically range from 10 µm to 200 μm, silica gel particles from 70 µm to 150 µm (Jesionowski et al., 2014), and calcium carbonate (CaCO_3_) particles generally range from 10 µm to 200 µm depending on preparation conditions (Boyjoo et al., 2014). CA beads prepared by dropwise extrusion were generally <1000 µm in diameter, with typical bead sizes of 200–800 µm reported for encapsulation applications (Lee et al., 2013).

### 2.7 Biochemical characteristics of free and immobilized PLC_S.a_

The effects of metal ions, temperature, pH, and bile salts on both free and immobilized PLC_S.a_ were examined by monitoring the hydrolysis of emulsified PC. Experiments were conducted over a temperature range of 30 °C–90 °C, pH 3.0–13.0, bile salt concentrations of 0–8 mM for NaDC and 0–6 mM for NaTDC, and varying concentrations of Ca^2+^, Mn^2+^, and Zn^2+^ (0–1 mM), all under optimal reaction conditions.

The storage stability of both free and immobilized PLCS.a was evaluated by measuring their enzymatic activity at 4 °C and 25 °C over a period of 90 days, with measurements taken every 10 days.

### 2.8 Biotechnological potential of PLC_S.a_ oil degumming

Free and immobilized PLC samples were tested for their efficiency in soybean oil degumming under optimized conditions (Zwaal et al., 1971). A mixture of soybean oil (200 mL) and citric acid (45%, 130 mL) was heated to 80 °C with stirring (900 rpm) for 5 min. After cooling to 60 °C, the pH was adjusted to 9.00 with 1 M NaOH, followed by the addition of 3 mL of water under gentle stirring (30 rpm) for 20 min. Subsequently, free or immobilized PLC (200 U/kg oil) was introduced, and the reaction was maintained at 60 °C with stirring (30 rpm) for 10 h. Samples were collected every hour for phosphorus analysis, centrifuged at 7000 × g to remove gums and immobilized enzymes, and the residual phosphorus in the oil phase was quantified using the AOCS method ca 12–55 (Society, 1990).

### 2.9 Reusability of immobilized PLC

In each reaction cycle, soybean oil was subjected to a degumming process using the recovered immobilized PLC. After the reaction, the biocatalyst was separated by centrifugation (9,000 × g for 5 min), washed three times with buffer A, and gently resuspended for reuse. The treated samples from each cycle were collected and analyzed as described in Section 2.8.

### 2.10 Statistical analysis

All statistical analyses were performed using GraphPad Prism. Data are expressed as mean ± standard deviation (SD) from at least three independent experiments. One-way ANOVA with Dunnett’s *post hoc* test was used to assess the differences between support materials. For the experiments with two factors (e.g., free vs. immobilized enzyme across different substrates or over storage time), a two-way ANOVA was used, followed by Tukey’s or Sidak’s *post hoc* test for multiple comparisons, as mentioned in the figure legends. Statistical significance was indicated as p < 0.05 (), p < 0.01 (*), p < 0.001 (*), and p < 0.0001 (**).

## 3 Results

### 3.1 Optimization of medium culture compounds for PLC production

#### 3.1.1 Carbon and nitrogen sources

**FIGURE 1:**
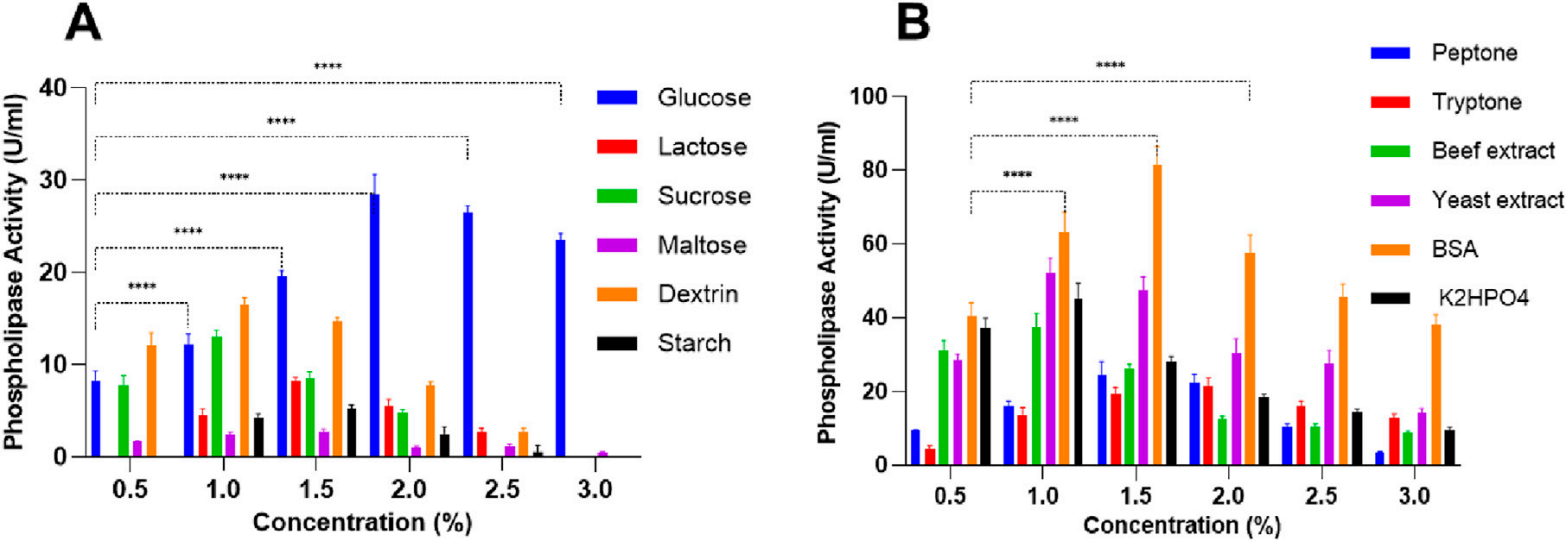
**(A)** Carbon and **(B)** nitrogen source impact on PLC activity in *Staphylococcus aureus* at 1% concentration. Data are expressed as the mean ± standard deviation from three separate experimental replicates. Statistical comparisons are shown only for glucose and BSA across different concentrations, as they were found to be the most effective inducers of PLC activity. Significance levels are indicated as: p < 0.0001 (****).

The influence of various carbon sources on PLC production in *S. aureus* revealed distinct patterns of activity depending on the type and concentration of sugar. Glucose was the most effective inducer, with PLC activity increasing from 8.25 U/mL at 0.5% to a maximum of 28.5 ± 2.12 U/mL at 2% (Figure 1A). After 2%, a decline was observed, suggesting catabolite repression (Troitzsch et al., 2021). Indeed, beyond providing cellular energy, glucose is known to influence *S. aureus* virulence by upregulating biofilm-related genes and modulating the expression of key virulence regulators as well as several toxins (Vitko et al., 2016). Similarly, dextrin supported moderate PLC production (16.5 ± 0.70 U/mL at 1%), followed by a progressive decrease (Figure 1A), which indicates that low dextrin levels are optimal for enzyme induction. Moreover, given that dextrin is composed of glucose polymers connected via glycosidic linkages, it likely influences *S. aureus* metabolism in a way comparable to glucose (Choueiry et al., 2022). Sucrose also promoted limited enzyme activity, with a maximum of 13 ± 0.70 U/mL at 1%, but higher concentrations led to complete inhibition (Figure 1A). Starch showed a weak inductive effect, with minimal activity detected only between 1% and 2% (maximum 5.25 ± 0.35 U/mL at 1.5%), and no activity at 0.5% and 3%, indicating low suitability for PLC production. In contrast, lactose and maltose were the least effective: lactose induced a modest peak of 8.25 ± 0.35 U/mL at 1.5%, while maltose showed minimal activity, reaching only 2.7 ± 0.28 U/mL (Figure 1A).

The effect of various nitrogen and phosphate sources on *S. aureus* PLC activity showed a concentration-dependent response, with different patterns observed for peptone, tryptone, beef extract, yeast extract, BSA, and K_2_HPO_4_ (Figure 1B). Peptone concentration led to a progressive increase in PLC activity, peaking at 24.5 ± 3.53 U/mL at 1.5% but declining at higher concentrations, reaching 3.25 ± 0.35 U/mL at 3%, pointing to an inhibitory effect at excessive concentrations (Figure 1B). Similarly, tryptone also demonstrated a concentration-dependent effect, with a PLC maximal activity of 21.5 ± 2.12 U/mL at 2% but dropping to 12.75 ± 1.06 U/mL at 3%, implying potential metabolic repression at higher concentrations (Figure 1B). Both beef extract and yeast extract showed an early rise in PLC activity. Beef extract peaked at 37.5 ± 3.53 U/mL when used at 1%, but its activity declined to 8.75 ± 0.35 U/mL at 3% (Figure 1B). Similarly, yeast extract reached a maximum of 52 ± 3.53 U/mL at 1%, followed by a drop to 14 ± 1.41 U/mL at the highest concentration tested. BSA was the most pronounced nitrogen source in increasing PLC activity, reaching 81.5 ± 4.94 U/mL at 1.5%, followed by a decrease to 38 ± 2.82 U/mL at 3%. K_2_HPO_4_ gradually increased PL activity from 37 ± 2.82 U/mL at 0.5% to 45 ± 4.24 U/mL at 1%, but activity dropped sharply to 9.5 ± 0.70 U/mL at 3%, following a pattern similar to other sources. (Figure 1B). These findings indicate that moderate concentrations of each nitrogen or phosphate source increase PLC production, but higher concentrations tend to inhibit enzyme activity, likely due to metabolic imbalances or nutrient excess.

#### 3.1.2 Effect of surfactants

**FIGURE 2:**
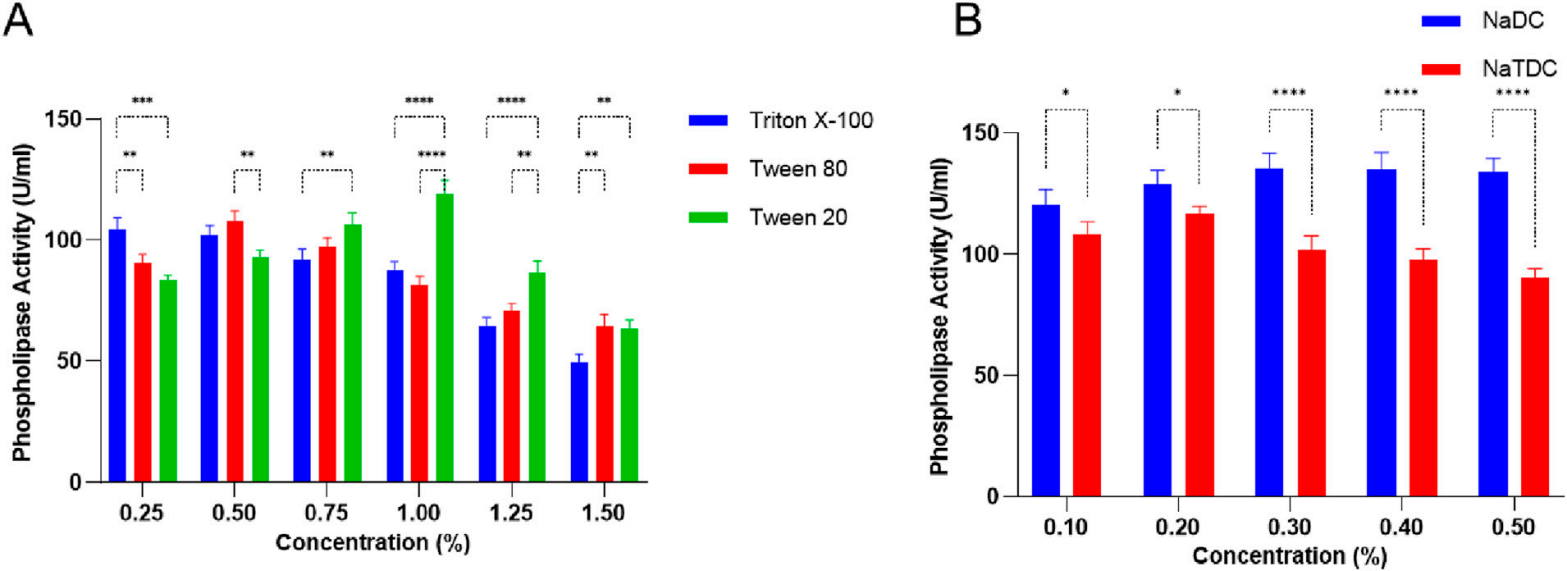
**(A)** Surfactants: Triton X-100, Tween 80, and Tween 20, and **(B)** bile salt impacts: sodium deoxycholate (NaDC) and sodium taurodeoxycholate (NaTDC) on PLC production by *Staphylococcus aureus*. Data are expressed as the mean ± standard deviation from three separate experimental replicates. Comparisons between detergents or bile salt at the same concentration significance levels are indicated as p < 0.05 (*), p < 0.01 (**), p < 0.001 (***), p < 0.0001 (****).

Different detergents and bile salts influence PLC activity in *S. aureus*, showing distinct patterns based on the additive type and concentration (Figure 2). Triton X-100 showed a noticeable concentration-dependent effect, where PLC activity stayed fairly high at lower doses (104.5 ± 4.94–102 ± 4.24 U/mL at 0.25%–0.5%) but dropped significantly to 49.5 ± 3.53 U/mL at 1.5% p < 0.0001 (Figure 2A). This decline may reflect enzyme instability or reduced substrate availability at increased surfactant levels. In contrast, Tween 80 and Tween 20 exhibited a biphasic response. PLC activity increased at low to moderate concentrations, reaching 108 ± 4.24 U/mL p *<* 0.01 at 0.5% and 119 ± 5.65 U/mL at 1%, respectively, probably due to improved enzyme-substrate contact or solubilization (Figure 2A). However, further increases in concentration led to reduced activity, possibly as a result of micelle formation or interference with enzyme function.

Among bile salts, sodium deoxycholate (NaDC) exhibited a strong and sustained stimulatory effect on PLC activity, peaking at 135.5 ± 6.63 U/g at 0.3% p < 0.0001 and maintaining high activity up to 0.5% (Figure 2B), which supports that it promotes favorable conditions for enzyme function. Conversely, sodium taurodeoxycholate (NaTDC) showed only a modest increase up to 117 ± 2.82 U/g at 0.2% before declining steadily, indicating that the enzyme is more sensitive to concentration changes, with marked inhibition at higher doses (Figure 2B). These findings indicate that non-ionic detergents such as Tween 20 and Tween 80 can promote PLC activity at moderate concentrations, whereas Triton X-100 exhibits a more suppressive effect.

#### 3.1.3 Effect of bivalent cations

The impact of various metal salts on PLC activity from *S. aureus* showed notable variations. ZnCl_2_ and CaCl_2_ significantly increased activity, reaching 168.5 ± 4.94 U/mL and 152 ± 2.82 U/mL, respectively, while MgCl_2_, MgSO_4_, and CuSO_4_ caused moderate increases in activity (128 ± 5.65 U/mL, 121.5 ± 4.94 U/mL, and 99.5 ± 4.94 U/mL, respectively) (Table 1). In contrast, BaCl_2_ and CoCl_2_ strongly inhibited the enzyme, with activities dropping to 73 ± 5.65 U/mL and 65.5 ± 3.53 U/mL, respectively, and BaSO_4_ and FeCl_3_ showed milder inhibitory effects. ZnSO_4_ also caused slight activation (101 ± 4.24 U/mL) (Table 1).

**TABLE 1:** Effect of various bivalent cations at 1% concentrations on *Staphylococcus aureus* PLC production. Data are expressed as the mean ± standard deviation from three separate experimental replicates. Comparisons were made for each cation treatment against the control (None). Significance levels: p < 0.01 (**), p < 0.0001 (****).

Bivalent cation (1% concentration)	PLC activity (U/mL)
None	107.5 ± 3.53
BaCl_2_	73 ± 5.65 ****
BaSO_4_	82 ± 4.24 **
CaCl_2_	152 ± 2.82 ****
COCl_2_	65.5 ± 3.53 ****
CuSO_4_	99.5 ± 4.94
MgCl_2_	128 ± 5.65 **
MgSO_4_	121.5 ± 4.94
FeCl_3_	86.5 ± 4.94 **
ZnSO_4_	101 ± 4.24
ZnCl_2_	168.5 ± 4.94 ****

**FIGURE 3:**
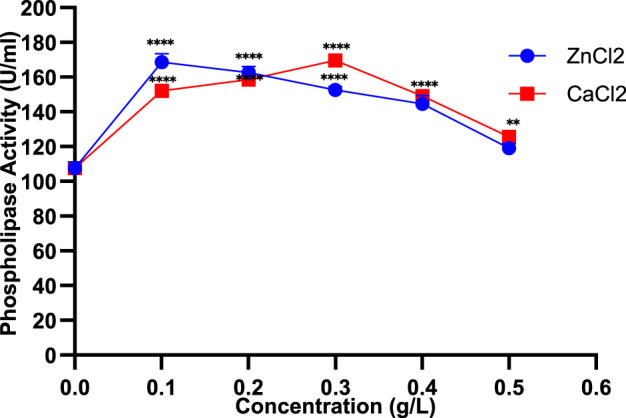
Impact of varying ZnCl_2_ and CaCl_2_ concentrations on PLC production in *Staphylococcus aureus*. Data are expressed as the mean ± standard deviation from three separate experimental replicates. Comparisons were performed for each concentration to the control (0 g/L). Significance levels are indicated as: p < 0.01 (**), p < 0.0001 (****).

Different concentrations of ZnCl_2_ demonstrated a dose-dependent response, with a marked increase in PLC activity observed at 0.1% (168.5 ± 4.94 U/mL), followed by a progressive reduction to 119 ± 2.82 U/mL at 0.5% (Figure 3). These results suggest that lower ZnCl_2_ levels may stimulate enzymatic activity, whereas higher concentrations exert an inhibitory effect. Similarly, increasing CaCl_2_ concentrations initially stimulated PLC activity, peaking at 169.5 ± 3.53 U/mL at 0.3%, but they showed a decline to 125.5 ± 3.53 U/mL at 0.5% (Figure 3). Collectively, divalent cations like Ca^2+^, Zn^2+^, and Mg^2+^ increase PLC activity, especially in chloride form, while barium and cobalt salts tend to suppress it.

### 3.2 Optimization of physicochemical factors influencing PLC production

#### 3.2.1 Incubation time

**FIGURE 4:**
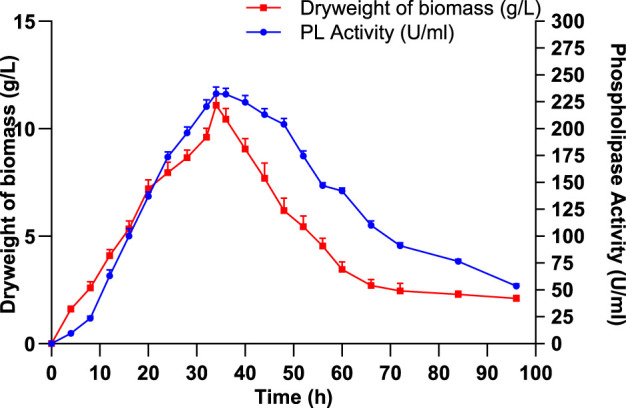
Impact of incubation duration on *Staphylococcus aureus* cell growth and PLC production. Data are expressed as the mean ± standard deviation from three separate experimental replicates.

In order to evaluate the relationship between enzyme synthesis and bacterial growth, the production of PLC and biomass accumulation by *S. aureus* was monitored during a 96-h fermentation period. Current data presented in Figure 4 show that both PLC activity and biomass increased progressively during the first 34 h, reaching their respective peaks at 232.5 ± 6.36 U/mL and 11.1 ± 0.84 g/L, indicating that PLC production is tightly linked to the exponential growth phase. This phase represents the period of most active metabolism, during which the bacterial cells are highly viable and actively producing extracellular enzymes (Gonzalez and Aranda, 2023). After 34 h, both PLC activity and biomass declined due to nutrient exhaustion, accumulation of toxic metabolites, or autolysis, leading the culture into the stationary and death phases. By 96 h, PLC activity had decreased to 53.5 ± 2.12 U/mL and biomass to 2.1 ± 0.14 g/L, indicating a significant reduction in bacterial viability and metabolism (Figure 4).

#### 3.2.2 Effect of pH and temperature

**FIGURE 5:**
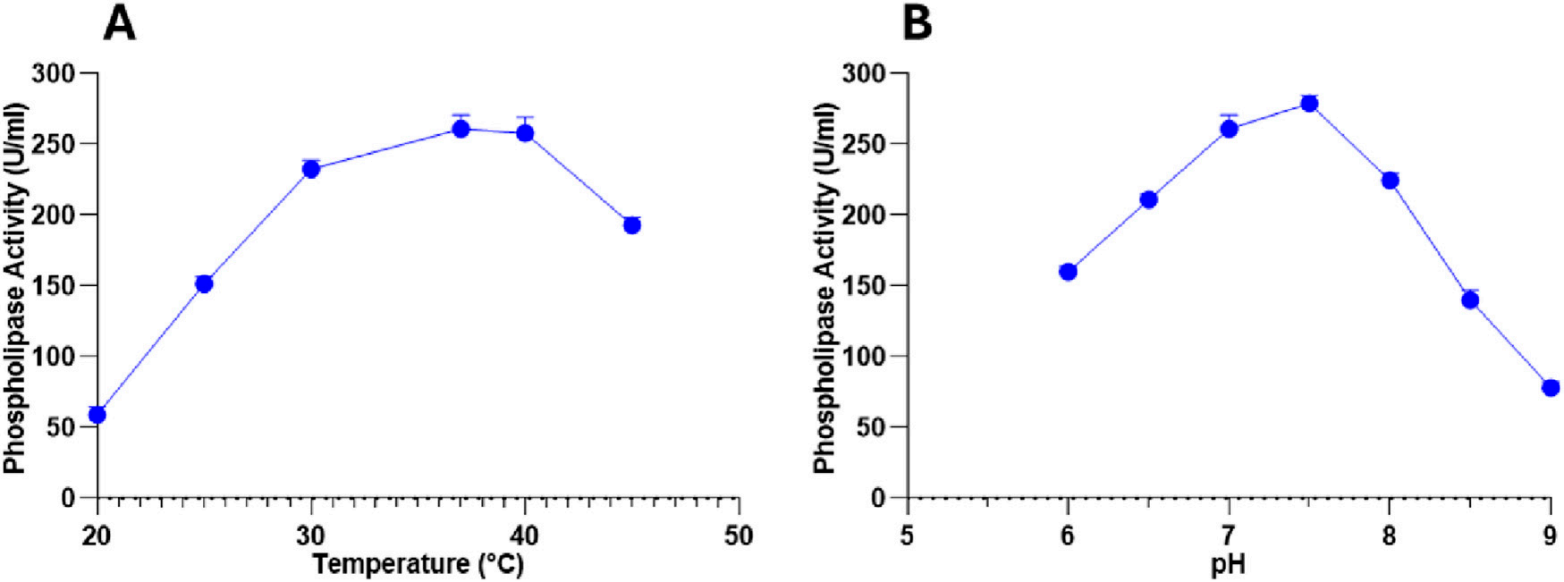
**(A)** Temperature and **(B)** pH impacts on PLC activity produced by *Staphylococcus aureus*. Data are expressed as the mean ± standard deviation from three separate experimental replicates.

To determine the optimal catalytic conditions, the PLC activity was evaluated under varying temperature and pH conditions. Temperature assays revealed a progressive increase in activity from 59 ± 5.65 U/mL at 20 °C to a peak of 261 ± 9.89 U/mL at 37 °C, corresponding to the enzyme’s optimal temperature (Figure 5A). Beyond 37 °C, PLC activity slightly decreased (258 ± 11.31 U/mL at 40 °C) and dropped further at 45 °C (193 ± 5.65 U/mL), probably due to a partial thermal denaturation at higher temperatures (Figure 5A).

Similarly, pH strongly influenced PLC activity, with a gradual rise from 160 ± 4.24 U/mL at pH 6.0 to a maximum of 279 ± 5.65 U/mL at pH 7.5, identifying this as the optimal pH for enzymatic function (Figure 5B). Activity declined significantly beyond this point, reaching 224.5 ± 4.94 U/mL at pH 8.0 and 78 ± 4.24 U/mL at pH 9.0 (Figure 5B). This result indicates that the enzyme exhibits optimal activity under slightly alkaline conditions and shows reduced efficiency when the pH shifts from the optimum.

### 3.3 Purification of PLC_S.a_

**FIGURE 6:**
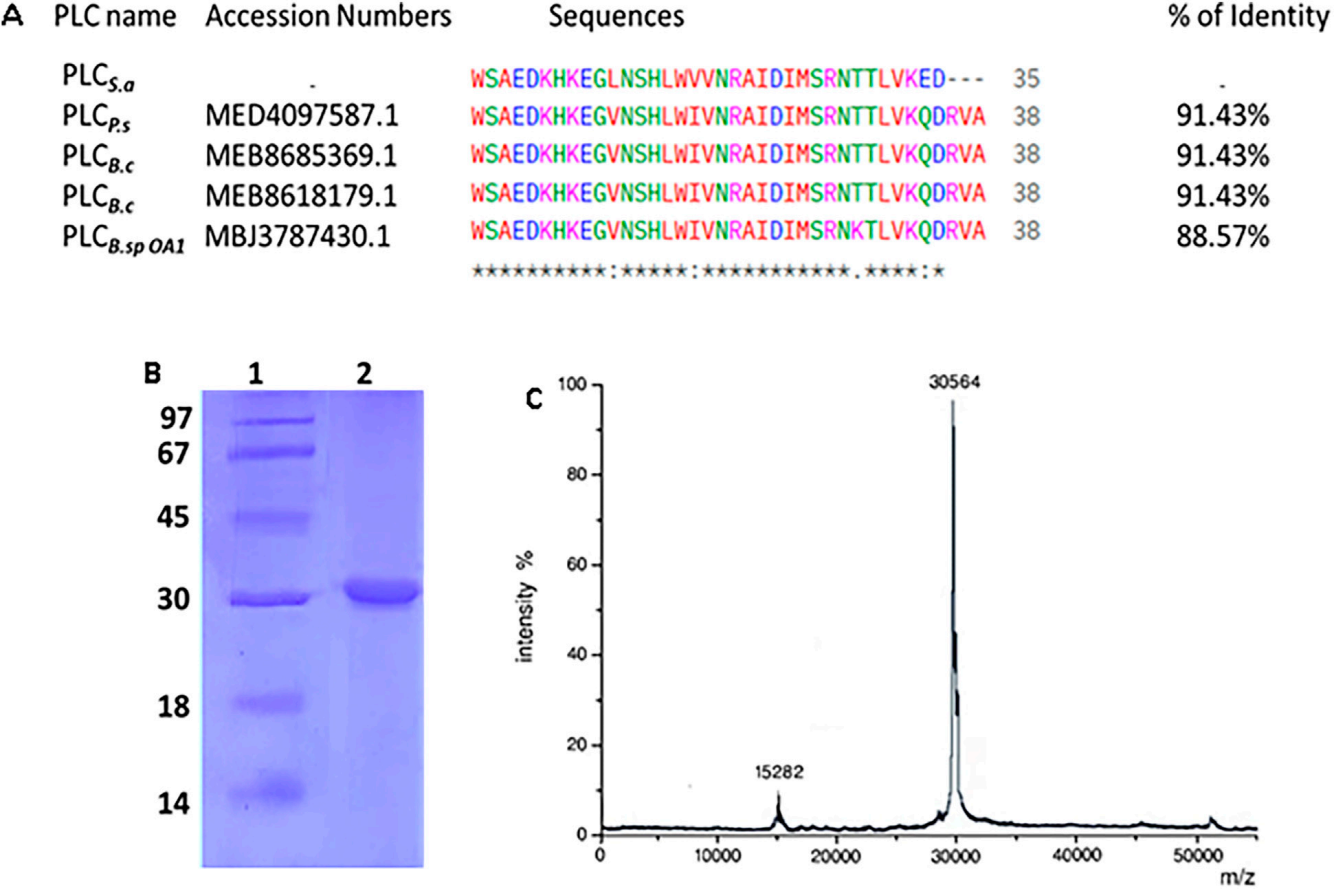
**(A)** NH_2_-terminal sequences alignment of purified PLC_S.a_ with other *Bacillus* PLCs. Amino acid sequence alignments were carried out using the program BLAST-P (NCBI, NIH, USA) database. (*) Highly conserved residues, (.) Non-conserved residues. **(B)** SDS-PAGE analysis of the purified PLC_S.a_. Lane 1: molecular mass markers, lane 2: purified PLC_S.a_. **(C)** MALDI-TOF spectra of PLC_S.a_.

The purification of PLC_S.a_ resulted in a progressive increase in specific activity and purification factor through each step. Starting from a crude extract with a total activity of 27,000 U and a specific activity (SA) of 172 U/mg, PLC_S.a_ was subjected to ammonium sulfate (NH_4_)_2_SO_4_ fractionation (30%–70%), which retained 76.26% of the activity and increased the specific activity to 467 U/mg (2.71-fold purification). Further purification using Sephadex G-75 gel filtration yielded 14,500 U of activity with a higher specific activity of 1,372 U/mg and a 53.7% recovery, corresponding to a 7.98-fold purification. Finally, the Mono Q-Sepharose ion exchange chromatography step resulted in 10,070 U of total activity with a specific activity of 2,850 U/mg, achieving a 37.3% overall recovery and a 16.56-fold purification, indicating a highly enriched PLC_S.a_ preparation (Table 2). SDS-PAGE analysis revealed a single, sharp band at approximately 30 kDa in Lane 2, corresponding to the purified PLC_S.a_ (Figure 6B). Consistently, MALDI-TOF mass spectrometry confirmed the molecular mass of the enzyme as 30 kDa (Figure 6C).

**TABLE 2:** Purification steps of PLC_S.a_

Purification steps	Total activity (U)	Protein (mg)	Specific activity (U/mg)	Activity recovery (%)	Purification factor
Extraction	27,000	157	172	100	1
(NH_4_)_2_ SO_4_ fractionation (30%–70%)	20,590	44.1	467	76.26	2.71
Sephadex G-75	14,500	10.57	1372	53.7	7.98
Mono Q-Sepharose	10,070	3.53	2850	37.3	16.56

The multiple sequence alignment of the N-terminal region of PLC_S.a_ with four related bacterial phospholipases revealed a high degree of conservation. The sequence shares 91% identity with PLC_P.s_, PLC_B.c1_, and PLC_B.c_, and 88.57% identity with PLC_B.spOA1_ (Figure 6A). Most residues are fully conserved across all sequences, particularly in regions likely involved in catalytic activity or substrate interaction. Minor differences are mainly located near the C-terminal end of the aligned region, where PLC_S.a_ shows a slightly shorter sequence.

### 3.4 Immobilization of PLC_S.a_

The immobilization yield of purified PLC_S.a_ varied notably across different support materials (Table 3). The highest efficiency was observed on CA combined with chitosan (CAC), reaching 82 ± 4.24%, followed by CA with glutaraldehyde (CAG) at 71.5 ± 6.63%, and CA alone at 61.5 ± 3.35%. In contrast, inorganic supports showed lower yields, with CaCO_3_ achieving 48 ± 4.24%, silica achieving 34 ± 4.24%, and Celite the lowest at 31 ± 2.82%.

**TABLE 3:** Immobilization yield of PLC on various support materials. The enzyme was immobilized on inorganic supports (CaCO_3_, Celite, silica) and calcium alginate-based carriers, including calcium alginate alone (CA), calcium alginate-chitosan (CAC), and calcium alginate-glutaraldehyde (CAG). Data are expressed as the mean ± standard deviation from three separate experimental replicates. Comparisons were made for CAC vs. other supports, as CAC gave the highest immobilization yield. Significance levels: p < 0.05 (*), p < 0.001 (***), p < 0.0001 (****).

Support material	Immobilization yield (%)
CaCO_3_	48 ± 4.24 ***
Celite	31 ± 2.82 ****
Silica	34 ± 4.24 ***
CA	61.5 ± 3.35 *
CAC	82 ± 4.24
CAG	71.5 ± 6.63

### 3.5 Biochemical characterization of free and immobilized PLC_S.a_: a comparative study

#### 3.5.1 Storage stability

**FIGURE 7:**
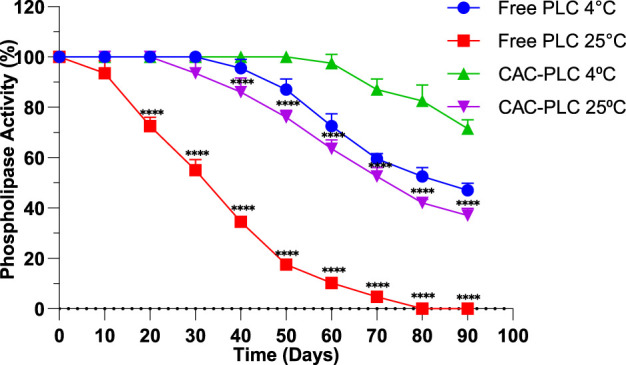
Storage stability of free and immobilized PLC: Percentage of activity retained at 4 °C and 25 °C. Values represent the mean ± standard deviation of three independent replicates. The activities of free and CAC-PLC were compared at each time point independently for the two storage temperatures to assess the effect of immobilization on enzyme stability. Significance levels are indicated as: p < 0.0001 (****).

The storage assay revealed that free PLC retained full activity for 20 days at 4 °C but gradually declined to 47 ± 2.82% activity by day 90. However, CAC-immobilized PLC remained fully active for 50 days and maintained 71.5 ± 3.53% activity at day 90 (Figure 7). This finding highlights the enhanced long-term stability conferred by immobilization. At 25 °C, free PLC activity dropped rapidly, reaching zero by day 80, whereas immobilized PLC stayed fully active for 20 days and retained 37 ± 2.82% activity at day 90 (Figure 7), demonstrating that CAC immobilization significantly improves stability even at room temperature.

#### 3.5.2 Effect of pH and temperature

**FIGURE 8:**
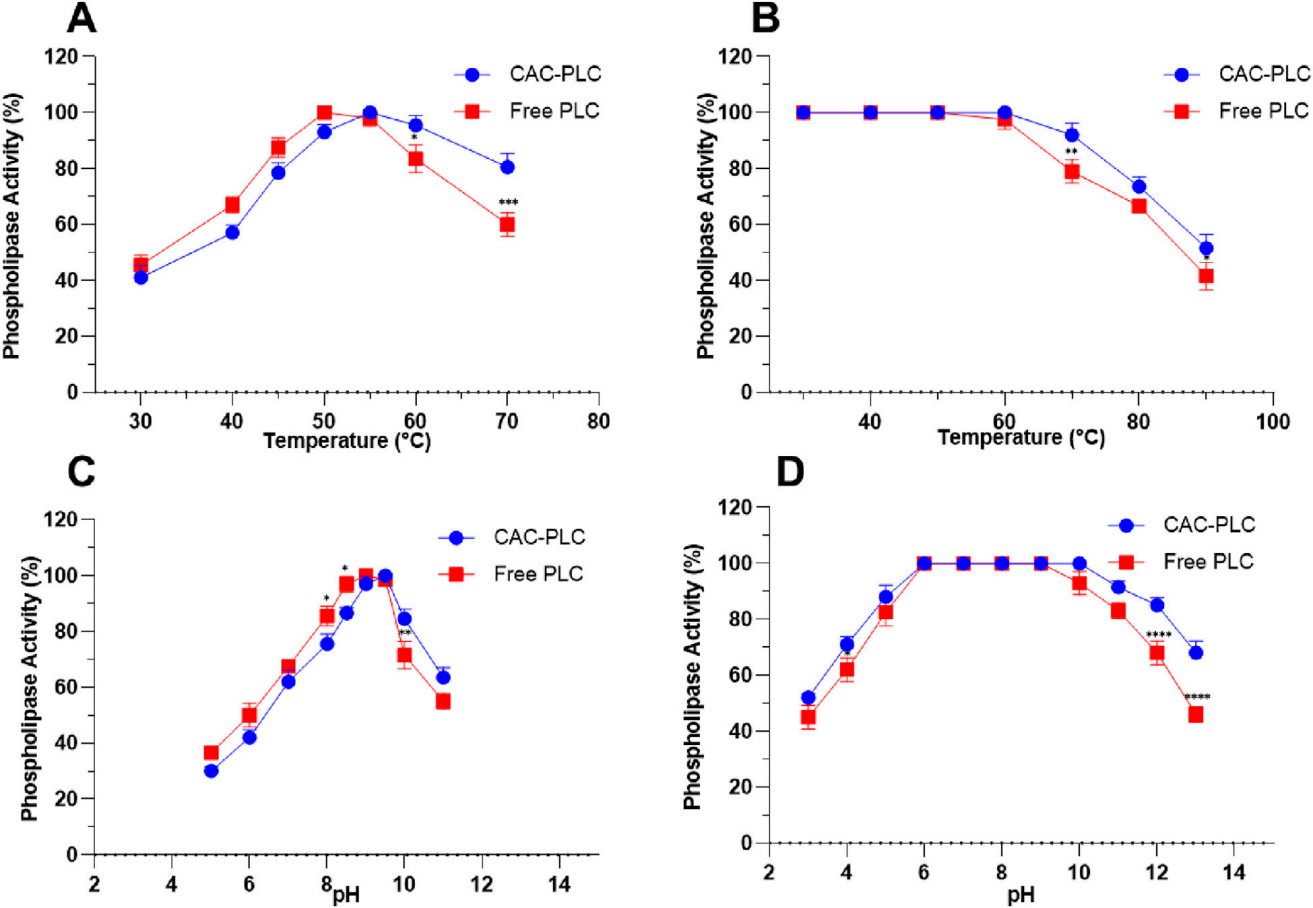
Effect of temperature and pH on free and immobilized PLC_S.a_ activity and stability. **(A)** Temperature-dependent activity of free and CAC-immobilized PLC. **(B)** pH-dependent activity of free and CAC-immobilized PLC. **(C)** Thermal stability of free and CAC-immobilized PLC over 30 °C–90 °C. **(D)** pH stability of free and CAC-immobilized PLC across pH 3.0–13.0. Data are expressed as the mean ± standard deviation from three separate experimental replicates. Comparisons were performed between free and CAC-PLC at each temperature and pH. Significance levels indicated as p < 0.1 (*), p < 0.01 (**), p < 0.001 (***), and p < 0.0001 (****).

The influence of temperature and pH on the activity of PLC, both free and immobilized on CAC, was investigated to evaluate the effect of immobilization on enzyme stability and performance (Figure 8). Regarding temperature, the free enzyme showed a gradual increase in activity from 45.5 ± 3.53 U/mL at 30 °C to a maximum of 100 ± 0 U/mL at 50 °C, followed by a decline at higher temperatures, reaching 60 ± 4.24 U/mL at 70 °C (Figure 8A). Immobilized CAC-PLC displayed a slightly shifted thermal profile, with activity rising from 41 ± 4.24 U/mL at 30 °C to a peak of 100 ± 0 U/mL at 55 °C and retaining 95.5 ± 3.53 U/mL at 60 °C.

Similarly, the effect of pH revealed that the free enzyme reached maximum activity (100 ± 0 U/mL) at pH 9.0, with notable decreases at higher pH values (55 ± 2.82 U/mL at pH 11.0) (Figure 8B). Immobilization on CAC shifted the optimal pH slightly toward alkaline conditions (100 ± 0 U/mL at pH 9.5) and maintained higher activity under elevated pH (84.5 ± 3.53 U/mL at pH 10.0 and 63.5 ± 3.53 U/mL at pH 11.0).

The stability of both free and immobilized PLC on CAC was evaluated in order to assess the impact of immobilization on enzyme performance under different temperatures (30 °C–90 °C) (Figure 8C) and pH values (3.0–13.0) (Figure 8D). The thermal stability of free and CAC-immobilized PLC was assessed after 1 h incubation at different temperatures (30–90 °C). Free PLC maintained full activity up to 50 °C, with a slight decrease at 60 °C (97.5 ± 3.53%), and showed progressive loss at higher temperatures (79 ± 4.24% at 70 °C and 41.5 ± 4.95% at 90 °C) (Figure 8C). In contrast, the immobilized enzyme displayed improved resistance, retaining 100% activity up to 60 °C and higher residual activity at elevated temperatures (92 ± 4.24% at 70 °C and 51.5 ± 4.95% at 90 °C) than the free PLC (Figure 8C). These findings indicate that CAC immobilization enhances PLC thermostability, particularly above 60 °C.

The pH stability of the two forms of PLC was assessed over the range 3.0–13.0 during a 1-h incubation (Figure 8D). Free PLC was stable between pH 6.0 and 9.0, retaining 100% activity, with partial activity at pH 5.0 (82.5 ± 4.95%) and pH 10.0 (93 ± 4.24%), but a marked decline was seen under extreme acidic or alkaline conditions (45 ± 4.24% at pH 3.0; 46 ± 2.82% at pH 13.0) (Figure 8D). Immobilization improved tolerance, as the enzyme maintained full activity from pH 6.0 to 10.0 and showed higher residual activity at extreme pH values (52 ± 1.41% at pH 3; 91.5 ± 2.12% at pH 11; 68 ± 4.24% at pH 13) (Figure 8D).

#### 3.5.3 Effect of bile salt and metal ions

**FIGURE 9:**
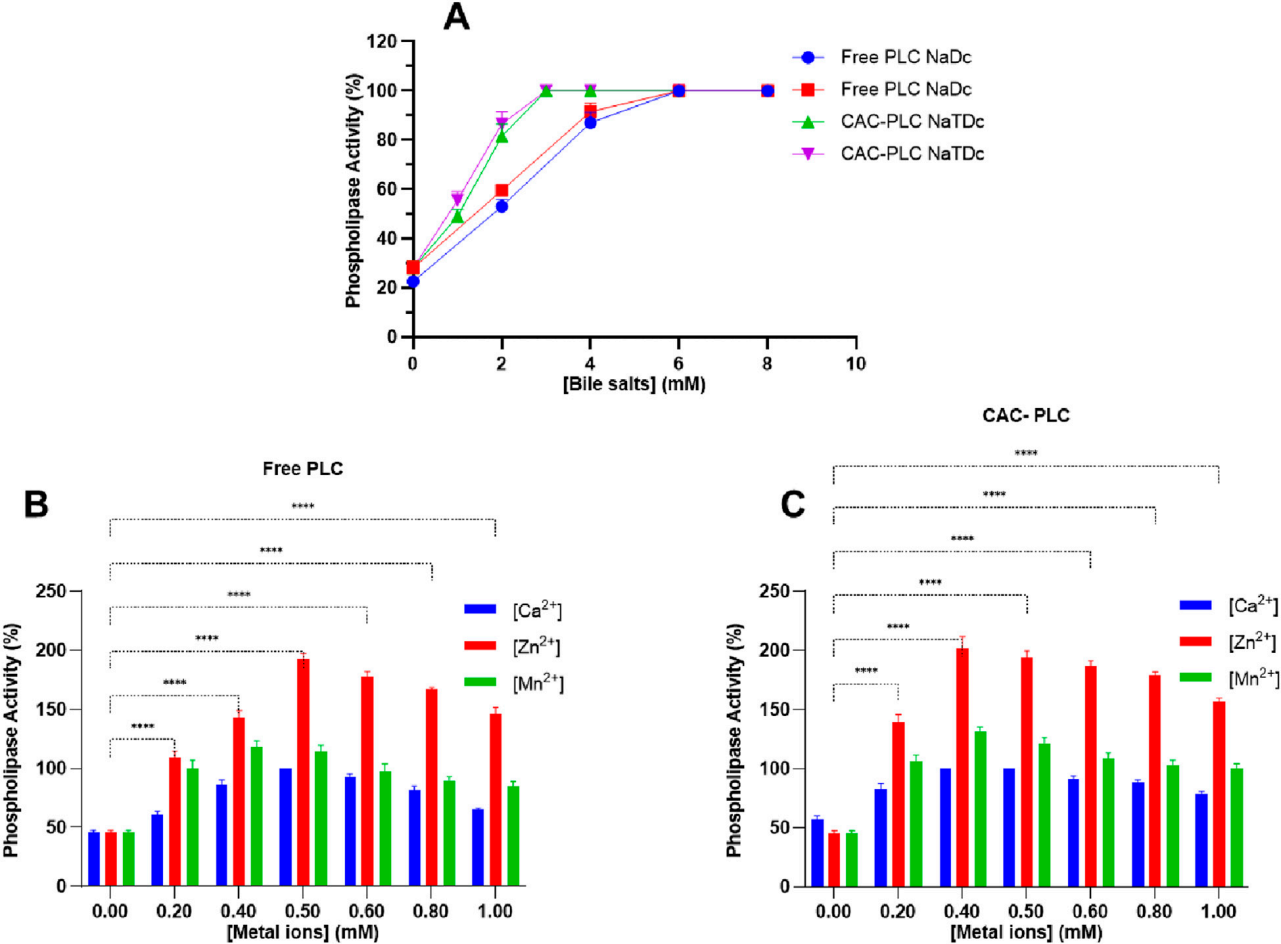
**(A)** Effect of bile salts on the activity of free and immobilized PLC (CAC-PLC). Effect of metal ions (Ca^2+^, Zn^2+^, and Mn^2+^) on the activity of **(B)** free and **(C)** immobilized CAC-PLC. Data are expressed as the mean ± standard deviation from three separate experimental replicates. Statistical comparisons are shown only for Zn^2+^, which exhibited the highest stimulatory effect on PLC activity. The absence of bars for Ca^2+^ and Mn^2+^ does not indicate a lack of significance.

The impact of various concentrations of bile salts on free and CAC-PLC activity was assessed. The activity of both free and immobilized PLC (CAC-PLC) increased in a concentration-dependent manner with bile salts. In the presence of NaDC (Figure 9A), activity rose from ∼23–28% at 0 mM to over 85% at 4 mM, reaching full activity (100%) at 6 mM, with no further increase at 8 mM. With NaTDC (Figure 9A), a similar profile was observed, but saturation occurred earlier, as activity increased sharply from 28% at 0 mM to >80% at 2 mM, and maximum activity (100%) was reached at 3 mM and maintained at higher concentrations.

The activity of both free and immobilized CAC-PLC was influenced by metal ions, each showing distinct optimal concentrations (Figure 9C). For free PLC, Ca^2+^ increased activity to 100 ± 0% at 0.5 mM, Zn^2+^ produced the strongest stimulation, reaching 192.5 ± 4.94% at 0.5 mM, and Mn^2+^ peaked at 118.5 ± 4.94% at 0.4 mM (Figure 9B). Similarly, immobilized CAC-PLC reached full activity with Ca^2+^ at 0.4–0.5 mM, exhibited maximal stimulation with Zn^2+^ at 202.5 ± 9.19% at 0.4 mM, and peaked at 131 ± 4.24% with Mn^2+^ at 0.4 mM (Figure 9C). In all cases, higher concentrations led to a gradual decline in activity, indicating that each metal has a specific optimal range for maximal enzyme activation, likely due to oversaturation or non-specific interactions with the enzyme. Indeed, excessive metal ions can disrupt the active site, induce structural perturbations, or cause competitive binding that interferes with catalysis, reducing enzymatic efficiency (Griffith and Ryan, 1999).

#### 3.5.4 Substrate specificity

Current results presented in Table 4 showed that both free and immobilized PLC exhibited broad substrate specificity, with the highest activity recorded toward phosphatidylcholine (PC, 100 ± 0%) (Table 4). For other phospholipids, immobilization generally increased activity compared to the free enzyme. In the case of PE, PG, and PS, CAC-PLC showed slightly higher hydrolysis rates (68.5 ± 4.94%, 45 ± 2.81%, and 52 ± 1.41%, respectively) relative to free PLC (60.5 ± 3.53%, 39 ± 2.82%, and 46 ± 1.41%, respectively). A similar trend was observed with sphingomyelin (63.5 ± 3.53% vs. 53.5 ± 4.94%) and phosphatidylinositol PI (26.5% vs. 20.5%). The most pronounced difference was found with cardiolipin, where CAC-PLC retained 23 ± 2.82% activity compared to only 11.75 ± 1.06% for free PLC, suggesting that immobilization improved substrate accessibility and catalytic efficiency across most phospholipid types.

**TABLE 4:** Comparative activity of free and immobilized phospholipase C (CAC-PLC) on different phospholipid substrates, including PC: phosphatidylcholine, PE: phosphatidylethanolamine, PG: phosphatidylglycerol, PS: phosphatidylserine, PI: phosphatidylinositol, sphingomyelin, and cardiolipin. Results are presented as a percentage of the activity measured on the reference substrate phosphatidylcholine (PC) under standard assay conditions. One unit of PLC activity corresponds to the production of 1 μmol of phosphocholine per minute. Data are expressed as the mean ± standard deviation from three separate experimental replicates. Comparisons were performed between free and immobilized phospholipase C with each substrate. Significance levels are indicated as p < 0.05 (*).

Phospholipid substrate	Phospholipase activity (%)	
	Free PLC	CAC-PLC
PC	100 ± 0	100 ± 0
PE	60.5 ± 6.53	68.5 ± 4.94
PG	39 ± 2.82	45 ± 2.82
PS	46 ± 1.41	52 ± 1.41
PI	20.5 ± 0.70	26.5 ± 0.70
Sphingomyelin	53.5 ± 4.94	63.5 ± 6.35
Cardiolipin	11.75 ± 1.06	23 ± 2.82 *

### 3.6 Soybean oil degumming using PLC_S.a_: biotechnological application

**FIGURE 10:**
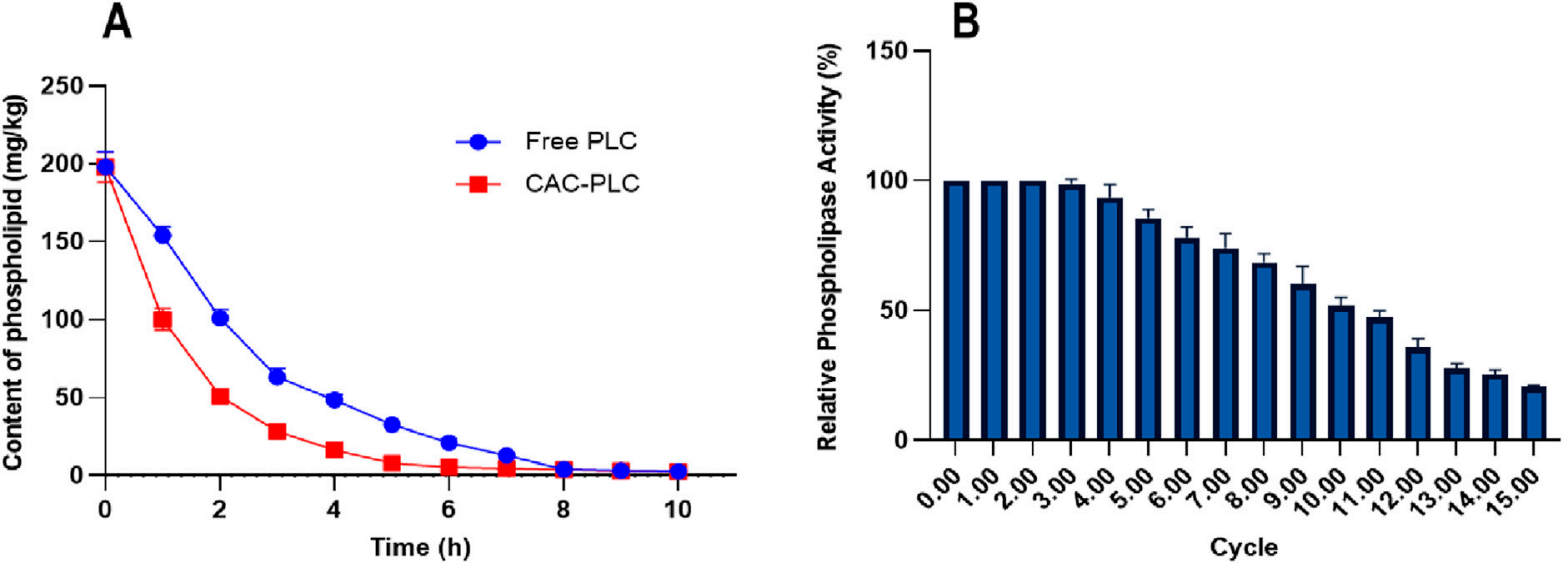
**(A)** Phosphorus content during soybean oil degumming by free and immobilized CAC-PLC. **(B)** Reusability of immobilized CAC-PLC in soybean oil degumming. Data are expressed as the mean ± standard deviation from three separate experimental replicates.

Given that PLC_S.a_ can hydrolyze various phospholipids, soybean oil was chosen to evaluate its degumming performance by monitoring both phosphorus and DAG contents (Figure 10A). The degumming of soybean oil was significantly accelerated by immobilization. Free PLC reduced phosphorus content gradually from 198 ± 9.89 mg/kg at 0 h to 2.5 mg/kg after 10 h, with notable decreases observed after 5–6 h (Figure 10A). In contrast, CAC-PLC achieved a faster reduction, reaching 51 ± 4.24 mg/kg at 2 h and 2.4 mg/kg by 10 h (Figure 10A). These results demonstrate that immobilization on CAC not only enhances the catalytic efficiency of PLC but also allows for more rapid and effective degumming of soybean oil than the free enzyme.

The reusability study of immobilized CAC-PLC during soybean oil degumming showed that the enzyme retained full activity up to the second cycle, with a slight decline to 98.5 ± 2.12% by the third cycle (Figure 10B). Progressive reductions in activity were observed thereafter, with relative activities of 93.5 ± 4.94% at the fourth cycle, 85.5 ± 3.53% at the fifth, and 78 ± 4.24% at the sixth (Figure 10B). Beyond the seventh cycle (74 ± 5.65%), activity gradually decreased, reaching 60 ± 7.07% at the ninth cycle and approximately half of the initial value (51.5 ± 3.53%) by the 10th (Figure 10B). The enzyme maintained moderate activity up to the 12th cycle (35.5 ± 3.53%) before showing a sharp decline, with only 20.5 ± 0.70% of its initial activity retained after 15 cycles. Current data highlight the good operational stability of CAC-PLC over multiple reuses despite gradual activity loss.

## 4 Discussion

**FIGURE 11:**
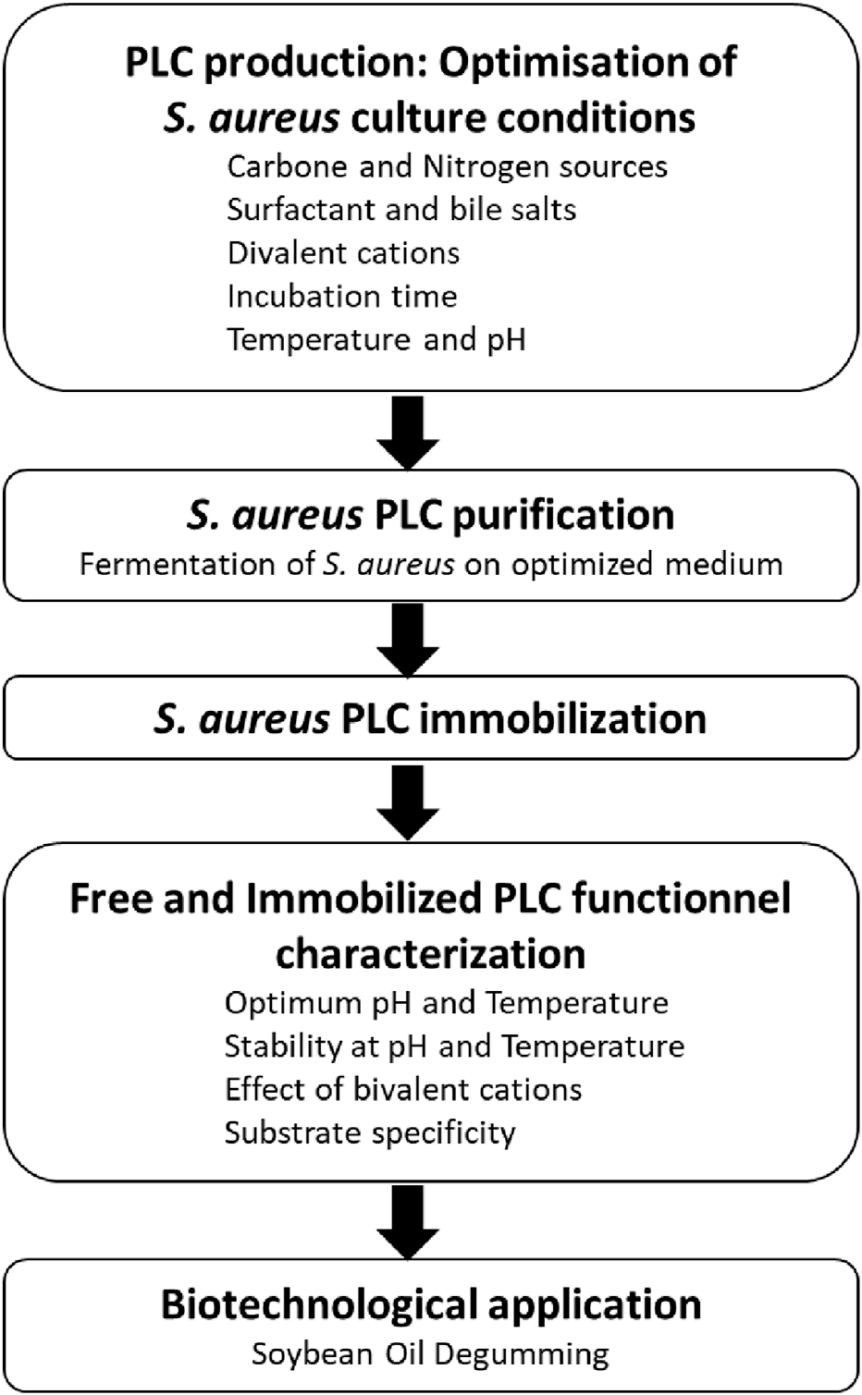
Schematic overview of PLC_S.a_ production, immobilization, and soybean oil degumming.

In this study, an extracellular PLC from *S. aureus* was optimized, purified, and immobilized for application in soybean oil degumming. To provide a clear overview of the experimental strategy and key steps undertaken, a schematic flowchart is presented in Figure 11. To optimize culture conditions for PLC production in *S. aureus*, the effects of different carbon, nitrogen, and phosphate sources were systematically evaluated. Current data showed that glucose and dextrin among carbon sources, and particularly BSA and yeast extract among nitrogen sources, were identified as the most effective in enhancing PLC production, although excessive concentrations of all tested compounds led to activity inhibition (Figure 1). The differences observed in PLC production with different carbon sources can be attributed to the interplay between sugar metabolism, carbon catabolite repression (CCR), and regulatory pathways controlling virulence in *S. aureus*. Readily metabolizable sugars such as glucose are known to strongly induce PLC expression at moderate concentrations, as they provide rapid energy and metabolic intermediates that increase enzyme synthesis. However, at higher concentrations, glucose triggers CCR, leading to repression of certain catabolic and virulence-associated genes, thereby explaining the decline in activity beyond 2% (Troitzsch et al., 2021). Other sugars like dextrin, which is composed of glucose polymers, can also moderately induce PLC because they are hydrolyzed to glucose monomers, feeding into similar metabolic and regulatory pathways (Choueiry et al., 2022). In contrast, disaccharides such as lactose and maltose, or polysaccharides like starch, require additional enzymatic processing before utilization. Their slower uptake and metabolism reduce available intracellular signals for virulence gene induction, resulting in weaker or negligible PLC expression (Somerville and Proctor, 2009). Similar findings were observed with bacterial PLC. Indeed, the production of PLC by *Pseudomonas fluorescens* MICAYA 2023 strains was significantly increased by the addition of soybean meal, yeast extract, NaCl, and egg yolk (Momtaz et al., 2025). Glucose (11 mM) and NH_4_Cl (5 mM) were used as the optimized carbon and nitrogen sources, respectively, for *Bacillus thuringiensis* (Elleboudy et al., 2016). Peptone (1%) and beef extract (0.5%) were used as the primary nitrogen sources for PLC production by *Bacillus mycoides* strain 970 (Wang et al., 2010). PLC production by the *Staphylococcus* isolate in a phosphate-starved medium was observed only when the glucose concentration was increased to 110 mM and BSA was added at concentrations of 0.5%, 1%, or 1.5% (Elleboudy et al., 2016). High yields of PLC from *S. aureus* D173 were achieved only when BSA was added to phosphate-starved TMM, with both growth and PLC production increasing progressively with higher BSA concentrations (Elleboudy et al., 2011).

The influence of different surfactants and bile salts on PLC activity in *S. aureus* was assessed to evaluate their potential modulatory effects. Tween 20, Tween 80 (Figure 2A), and sodium deoxycholate (Figure 2B) increased PLC activity at moderate concentrations, while Triton X-100 and sodium taurodeoxycholate showed inhibitory effects at higher doses. In *S. aureus* D173, all tested surfactants slightly increased growth except Tween 20, which significantly increased growth at 0.125% and 0.25% (w/v), while only Tween 20 supported minimal PLC production at these concentrations, unlike Triton X-100, Tween 80, and sodium cholate, which showed no effect (Elleboudy et al., 2011).

The effect of different divalent cations on *S. aureus* PLC activity was investigated to determine their stimulatory or inhibitory roles. Zn^2+^ and Ca^2+^ markedly increased PLC activity at low to moderate concentrations (Figure 3), Mg^2+^ produced moderate stimulation, while Ba^2+^ and Co^2+^ salts exhibited a strong inhibitory effect (Table 1). This differential response is likely due to the specific interactions of these metals with the enzyme’s active site: Zn^2+^ and Ca^2+^ act as cofactors stabilizing the catalytic center and promoting hydrolysis (Goldstein et al., 2012), Mg^2+^ partially substitutes in the active site or modulates enzyme conformation (Goldstein et al., 2012), whereas Ba^2+^ and Co^2+^ are incompatible with active site geometry, causing structural perturbations that inhibit activity (Hough et al., 1989; Little et al., 1982). Similarly, calcium carbonate (0.55 g/L) served as the bivalent cation source to increase PLC activity in *P. fluorescens* MICAYA 2023 strain (Momtaz et al., 2025). In fact, several PLCs produced by *Bacillus* strains require a millimolar concentration of Mg^2+^, Zn^2+^, or Ca^2+^. The highest PLC production from *S. aureus* D183 was observed when K_2_SO_4_ was replaced with Na_2_SO_4_, while the combined removal of CaCl_2_, MgCl_2_, and K_2_SO_4_ had no enhancing effect. This highlights that specific salts can modulate enzyme synthesis, possibly by affecting ionic balance or cofactor availability in the growth medium (Elleboudy et al., 2011).

To investigate the relationship between enzyme synthesis and bacterial growth, PLC production and biomass accumulation were monitored over a 96-h fermentation period. Both PLC activity and biomass reached their maximum at 34 h before progressively declining, indicating that enzyme production was closely associated with the exponential growth phase (Figure 4). The timing of maximal PLC production varies among bacterial strains and is influenced by several factors, including strain-specific metabolic rates, growth kinetics, environmental adaptations, genetic regulation, and culture conditions. Indeed, it is widely described that the incubation period, which can range from 24 h to a week depending on the type of microbe and culture conditions, has a significant impact on the production of microbial enzymes (Contesini et al., 2018). The effect of incubation period on PLC production by *P. fluorescens* MICAYA at 28 °C during 72 h showed maximum activity of 53.03 U/mL at 20 h (Momtaz et al., 2025). PLC from *B. mycoides* strain 970 (*B. mycoides)* and *B. thuringiensis (B. thuringiensis)* recovered from Egyptian soil were cultured until maximum activity after 20 h and 24 h, respectively (Wang et al., 2010; Elleboudy et al., 2016). For *S. aureus* D173, growth peaked at 24 h and declined after 48 h, while PLC production began at 24 h, reaching its maximum at 36 h (Elleboudy et al., 2011).

The catalytic behavior of PLC was evaluated under different temperature and pH conditions to identify the optimal parameters for enzyme activity. Maximum PLC activity was observed at 37 °C (Figure 5A) and pH 7.5 (Figure 5B), with reduced activity recorded at higher temperatures and at more acidic or alkaline pH values. These findings highlight that *S. aureus* PLC exhibits the highest activity at 37 °C and pH 7.5 conditions that align with the human host environment, supporting its potential role in virulence (Khelissa et al., 2017). Similarly, *B. mycoides* strain 970 and *B. thuringiensis* displayed maximal PLC activity at 30 °C and pH 7.0 (Wang et al., 2010; Elleboudy et al., 2016). Likewise, PLC from *P. fluorescens* MICAYA 2023 presented the highest enzymatic activity at pH 6.5 and 48.5 °C (Momtaz et al., 2025). *S. aureus* D173 showed considerable growth and PLC production only at temperatures of 25 °C, 30 °C, and 37 °C (Elleboudy et al., 2011). Table 5 summarizes the optimal culture and physicochemical conditions for PLC_S.a_ production along with the corresponding enzyme activities. These results highlight the key factors influencing PLC yield and provide a basis for future optimization using multivariate approaches such as response surface methodology (RSM).

**TABLE 5:** Summary of optimum conditions for PLC production by *Staphylococcus aureus*.

Parameter	Optimum condition	PLC activity (U/mL)
Carbon source	Glucose 2%	28.5 ± 2.12
Nitrogen source	BSA 1.5%	81.5 ± 4.94
Phosphate source	K_2_HPO_4_ 1%	45 ± 4.24
Surfactant	Tween 20 1%	119 ± 5.65
Bile salt	NaDC 0.3%	135.5 ± 6.63
Divalent cation	ZnCl2 0.1%	168.5 ± 4.94
Incubation time	34 h	232.5 ± 6.36
Temperature	37 °C	261 ± 9.89
pH	7.5	279 ± 5.65

To assess the biochemical properties of PLC_S.a_, the enzyme was purified through successive chromatographic steps, leading to a progressive enrichment in specific activity and purification fold. The stepwise purification strategy yielded a highly enriched PLC_S.a_ preparation (16.56-fold purification, 37.3% recovery), confirming the efficiency of the protocol and allowing reliable biochemical and functional characterization (Table 2). SDS-PAGE analysis revealed a single, sharp band at approximately 30 kDa (Figure 6B), confirmed with MALDI-TOF spectra (Figure 6C). This molecular mass was slightly lower than that of the recombinant PI-PLC from *S. aureus* with 32 KDa (Daugherty and Low, 1993) and slightly higher than several previously reported recombinant PLCs, including those from *B. cereus* (Pomerantsev et al., 2003) and *B. thuringiensis* (Elleboudy et al., 2016) with 28 KDa. However, it was much lower than the PLC from *B. mycoides* strain 970, which has a molecular mass of 75.1 kDa (Wang et al., 2010).

For enzymes to perform efficiently in industrial settings, stability must be improved. The stabilization approach and support material depend on the intended application. Common strategies include immobilization, protein engineering, chemical modification, and soluble additives. Of these, immobilization is most widely used because it enhances enzyme reuse, tolerance to harsh conditions, and simplifies recovery from reaction mixtures (Wahab, 2025). The immobilization of purified PLC_S.a_ was most efficient on biopolymeric supports, particularly CAC (82%), whereas inorganic carriers such as CaCO_3_, silica, and Celite exhibited markedly lower yields (48%–31%) (Table 3). These results are consistent with the well-documented effects of alginate, a versatile biopolymer with favorable physical and chemical properties for protein binding. Alginate-based immobilization is known to increase catalytic activity and stability with minimal drawbacks, owing to its specific interactions with proteins, which are influenced by ionic strength, pH, and metal ions (Chaudhari et al., 2015). However, the use of CA alone is often limited by biomolecule leakage, low mechanical strength, and relatively large pore size (Zhou et al., 2010). Various modifications have been developed to overcome these limitations, including covalent crosslinking with polymers such as chitosan or polyacrylic acid, and surface coating with reagents like poly-L-lysine or glutaraldehyde (Zhou et al., 2010). In agreement with these reports, our results show that CAC and CAG achieved higher immobilization efficiencies than CA alone.

Enzyme immobilization represents an effective strategy for tailoring and improving biocatalytic performance. It can influence key characteristics such as catalytic efficiency, substrate specificity, tolerance to fluctuations in pH and temperature, and the ability to be reused over successive reaction cycles (Hussain et al., 2023). These advantages are especially apparent in applied contexts, as reflected by the enhanced storage stability observed for immobilized PLC compared to its free form (Figure 7). CAC-immobilized PLC exhibited significantly enhanced storage stability compared to the free enzyme, maintaining higher activity over prolonged periods at both 4 °C and 25 °C (Figure 7). Similarly, numerous studies have shown that immobilization is a reliable approach for maintaining enzyme activity and substantially extending shelf life, thereby providing more consistent and durable performance in catalytic processes (Cavalcante et al., 2021).

Addressing industrial demands for higher productivity and longer shelf life, enzyme immobilization increases substrate availability and turnover over extended periods while improving activity and stability across a broader pH and temperature range, supporting large-scale, cost-effective applications (Khan et al., 2021). In this line, the effects of temperature and pH on the activity of both free and CAC-immobilized PLC were examined to assess how immobilization influences enzyme stability and catalytic performance (Figure 8). Immobilization on CAC slightly shifted the optimal temperature and pH of PLC and increased its activity under higher temperatures and more alkaline conditions compared to the free enzyme (Figures 8A,B). These results indicate that CAC-PLC not only slightly shifts the optimal conditions for PLC activity but also significantly enhances enzyme stability under both high temperature and alkaline conditions. Similar results were reported for PLC_BC_ from *B. cereus* (CICC-21688), where immobilization shifted the enzyme optima from pH 7.0 to 7.5 and from 55 °C to 60 °C, indicating increased stability (Yu et al., 2022). Yu et al. explained this stabilization by ionic interactions between negatively charged enzyme groups and positively charged carrier groups. Immobilization on the magnetic carrier further preserved PLC_BC_ conformation, broadened its optimal temperature range, and enhanced heat resistance through multipoint covalent bonding (Liu et al., 2019). CAC immobilization significantly enhanced the thermal and pH stability of PLC, allowing the enzyme to retain higher activity at increased temperatures and more extreme pH values than the free form (Figures 8C,D). Current data indicate that immobilization on CAC slightly shifts the enzyme’s optimal temperature and pH toward higher values and markedly enhances its stability under thermal and pH stress compared to the free enzyme. This aligns with previous studies showing that immobilization modifies enzyme properties through structural changes and the physicochemical nature of the support, thereby affecting enzyme function and applications (Khan et al., 2021).

The immobilization performance of phospholipases is strongly influenced by support characteristics such as pore size and surface architecture. Large pore diameters facilitate enzyme entry and reduce steric hindrance, which otherwise limits substrate accessibility and catalytic turnover. Conversely, supports with small or irregular pores may restrict conformational mobility of the enzyme, leading to reduced activity or stability. This observation is consistent with previous studies showing that pore size critically determines the performance of immobilized enzymes (Bayne et al., 2013) and that mesoporous silica nanospheres with optimized pore architecture enhance enzyme accessibility and catalytic efficiency (Kothalawala et al., 2022). In our case, the relatively high activity recovery observed after immobilization suggested that the support provided sufficient porosity and minimal steric restriction to maintain catalytic function.

Bile salts, acting as amphipathic detergents, enhance PLC activity by improving substrate accessibility and facilitating catalytic turnover, an effect that is particularly valuable for industrial applications such as enzymatic oil degumming, where increased enzyme efficiency leads to cleaner and more sustainable vegetable oil refining processes (Alonazi et al., 2023). Both free and CAC-immobilized PLC exhibited a concentration-dependent increase in activity with bile salts, achieving full activity at lower concentrations with NaTDC than with NaDC (Figure 9A). The rise in PLC activity with increasing bile salt concentrations can be attributed to detergent-induced conformational changes that favor a more active enzyme state. In addition, bile salts enhance substrate accessibility by forming mixed micelles and reducing product inhibition, collectively facilitating faster hydrolysis (El-Sayed and Roberts, 1985).

Regarding the bivalent cation effect, both free and CAC-immobilized PLC were strongly activated by Ca^2+^, Zn^2+^, and Mn^2+^ at specific optimal concentrations, with Zn^2+^ producing the highest stimulation, while higher concentrations of all metals led to decreased activity (Figures 9B,C). A similar behavior has been reported for PLC from *B. mycoides* strain 970; its activity was strongly dependent on Ca^2+^, Ba^2+^, Zn^2+^, Mn^2+^, and Mg^2+^ (Wang et al., 2010). These findings are in line with the described bacterial PLCs that are classified among metalloenzymes, with a tri-metal zinc center that is critical for efficient phospholipid hydrolysis (Lyu et al., 2016). Indeed, the crystal structure of B. cereus PLC_BC_ reveals that its active site contains three zinc ions, positioned in close proximity to residues essential for catalysis (Hough et al., 1989). Enzyme activity reaches its maximum when each protein molecule binds two or three Zn^2+^ ions. Furthermore, studies have shown that other divalent cations, such as Mg^2+^ or Ca^2+^, can substitute for zinc in the active site during catalysis, influencing hydrolysis efficiency (Hansen et al., 1993).

The substrate specificity of PLC is crucial for its application in enzymatic degumming of vegetable oils, where it efficiently hydrolyzes phospholipids such as PC, PE, and PS, leading to improved oil yield and quality (Lyu et al., 2016). Both free and CAC-immobilized PLC displayed broad substrate specificity, with immobilization generally increasing hydrolysis rates across most phospholipids, particularly cardiolipin (Table 4). Despite the observed improvements in activity, the substrate specificity of the immobilized CAC-PLC remained largely unchanged. It can be due to the fact that immobilization primarily affects the enzyme’s stability and catalytic efficiency rather than its substrate recognition (Khan et al., 2021). Although broad substrate specificity has been reported for PLCs from *Bacillus* species and other bacterial strains, which efficiently hydrolyze phospholipids such as PC, PE, PG, and PS (Alonazi et al., 2023), the PLC from *S. aureus* has been described as highly specific for phosphatidylinositol (PI), functioning essentially as a PI-PLC (Daugherty and Low, 1993). This result highlights the diversity in substrate preferences among bacterial PLCs and may reflect differences in physiological roles or biotechnological applications. In 2012, the crystal structure of *S. aureus* PI-PLC (strain FPR3757) revealed that the enzyme is highly active toward PI/PC vesicles but not pure PI. Its activity is associated with a significant conformational change in the rim mobile loop, driven by a titratable π–cation interaction involving histidine. Such interactions, commonly observed in proteins, contribute to enzyme stability and function (Goldstein et al., 2012).

Phospholipids in crude vegetable oils can cause dark coloration and the development of off-flavors during storage, making their removal a critical step for producing high-quality oil with phosphorus levels below 10 mg/kg (Borrelli and Trono, 2015). In industrial oil refining, hydratable phospholipids are typically removed by water degumming, while non-hydratable forms require chemical or enzymatic treatments, most commonly using phospholipases. Enzymatic degumming offers several advantages, including higher oil yield, reduced use of acids and alkalis, lower wastewater production, minimized environmental impact, and decreased operating costs (Bora, 2013). Among phospholipases, PLCs are particularly effective, generating oil-soluble sn-1,2-diacylglycerols (DAGs) with nutritional value and water-soluble organophosphates (Elleboudy et al., 2016). Soybean oil, the most widely consumed vegetable oil, contains a diverse mixture of lysophospholipids, PC, PI, DAGs, TAGs, and glycolipids (Eddehech et al., 2023). CAC-immobilized PLC significantly accelerated soybean oil degumming compared to the free enzyme, achieving near-complete phosphorus removal within 10 h (Figure 10A), and demonstrated good operational stability by retaining substantial activity over multiple reuse cycles, with more than 50% activity maintained even after ten cycles (Figure 10C). Several studies reported the efficiency of free bacterial PLC in degumming oil, especially from *Bacillus* strains. Indeed, purified PLC from *B. stearothermophilus* efficiently reduced phosphorus content in soybean oil to 35 mg/kg while simultaneously increasing the release of diacylglycerols (Alonazi et al., 2023). The recombinant PI-PLC enzyme from the *B. thuringiensis* decreased the phosphorus content of soybean oil down to 30.87 mg/kg (Eddehech et al., 2023). Moreover, oil degumming experiments showed that a double mutant of PLC from *Talaromyces islandicus* outperformed the wild-type, reducing residual phosphorus to 78 ppm compared to 131 ppm (Tang et al., 2025). A recombinant PI-PLC from *L. sphaericus*, produced at high yield (∼14 g/L) in *E. coli*, efficiently hydrolyzed crude oil phospholipids. Pilot-scale degumming confirmed by NMR provided an extra 2.13% oil yield compared to aqueous methods, highlighting its feasibility as a cost-effective process for industrial oil refining (Cerminati et al., 2017). TtPLC from *Bacillus licheniformis* MTCC 7445 effectively reduced residual phosphorus in crude soybean oil from 135.4 mg/kg to 7.9 mg/kg under optimized conditions, meeting the standards for environmentally friendly physical refining (Xiang et al., 2020).

Compared to bacterial PLCs from *Bacillus* and *Pseudomonas*, the PLC_S. a_ provided unique value by combining biomedical and biotechnological relevance. Unlike *Bacillus* PLCs, which typically display broader phospholipid specificity and higher thermal tolerance, PLC_S.a_ is essentially a PI-specific enzyme that operates optimally at 37 °C and pH 7.5, conditions that mirror the human host environment. This dual alignment with virulence and physiological conditions makes it of particular interest for therapeutic studies as well as industrial use. In addition, while PLCs from *Bacillus* and *Pseudomonas* strains are well known for their role in enzymatic oil degumming, the present work shows that immobilized PLC_S.a_ achieves rapid phosphorus removal and retains more than 50% activity after ten reuse cycles, highlighting good operational stability. These findings indicate that PLC_S.a_ can perform comparably to established bacterial PLCs in biocatalytic processes, while also offering added value as a medically relevant enzyme, thus expanding the repertoire of microbial PLCs available for both industrial and biomedical applications.

## 5 Conclusion

This study demonstrated that immobilization of PLC_S.a_ on CAC significantly increased its stability, catalytic efficiency, and reusability for soybean oil degumming. The immobilized enzyme efficiently reduced phosphorus content to below industrial standards and retained substantial activity over multiple reuse cycles, confirming its potential as a cost-effective and environmentally sustainable alternative for large-scale vegetable oil refining. These findings underscore the industrial relevance of immobilized PLC_S.a_, offering a promising strategy to improve oil yield while minimizing chemical usage and wastewater generation. Future perspectives include scaling up the process, further optimization using multivariate approaches such as RSM, and exploring other microbial PLCs to expand the toolbox of enzymes suitable for industrial applications.

## Data Availability

The datasets presented in this study can be found in online repositories. The names of the repository/repositories and accession number(s) can be found in the article/supplementary material.
